# The Use of Multiplicity Corrections, Order Statistics and Generalized Family-Wise Statistics with Application to Genome-Wide Studies

**DOI:** 10.1371/journal.pone.0154472

**Published:** 2016-04-29

**Authors:** Steven J. Schrodi

**Affiliations:** 1 Center for Human Genetics, Marshfield Clinic Research Foundation, Marshfield, WI, United States of America; 2 Computation and Informatics in Biology and Medicine, University of Wisconsin-Madison, Madison, WI, United States of America; Indiana University Bloomington, UNITED STATES

## Abstract

The most important decision faced by large-scale studies, such as those presently encountered in human genetics, is to distinguish between those tests that are true positives from those that are not. In the context of genetics, this entails the determination of genetic markers that actually underlie medically-relevant phenotypes from a vast number of makers typically interrogated in genome-wide studies. A critical part of these decisions relies on the appropriate statistical assessment of data obtained from tests across numerous markers. Several methods have been developed to aid with such analyses, with family-wise approaches, such as the Bonferroni and Dunn-Šidàk corrections, being popular. Conditions that motivate the use of family-wise corrections are explored. Although simple to implement, one major limitation of these approaches is that they assume that p-values are i.i.d. uniformly distributed under the null hypothesis. However, several factors may violate this assumption in genome-wide studies including effects from confounding by population stratification, the presence of related individuals, the correlational structure among genetic markers, and the use of limiting distributions for test statistics. Even after adjustment for such effects, the distribution of *p*-values can substantially depart from a uniform distribution under the null hypothesis. In this work, I present a decision theory for the use of family-wise corrections for multiplicity and a generalization of the Dunn-Šidàk correction that relaxes the assumption of uniformly-distributed null p-values. The independence assumption is also relaxed and handled through calculating the effective number of independent tests. I also explicitly show the relationship between order statistics and family-wise correction procedures. This generalization may be applicable to multiplicity problems outside of genomics.

## Introduction

A recurring question in large-scale studies concerns the proper statistical treatment of findings when numerous tests are performed. Using nominal significance levels as a threshold for reporting findings is prone to false positives. Historically, this is particularly problematic in genetic epidemiology studies focused on disease association within sets of candidate genes. It is well-known that the rate of replicating results from such studies was exceedingly low, suggesting ubiquitous type I errors [1,2]. Several shortcomings likely conspired to produce high type I error rates for these studies including publication bias, population stratification, reporting of the most significant result following the use of a number of analysis methods and tests, and the previous frequent use of a nominal α = 0.05. The era of genome-wide association studies has greatly benefited from the widespread adoption of experiment-wise significance levels which has dramatically improved the replication of positive findings [3,4].

In some instances—but not all—it is useful to think of interpretation of reported results as being dependent on the total number of tests performed as part of a study. Numerous methods have been devised to address this multiplicity problem and most provide a fairly reasonable tradeoff between false positive and false negative error rates across many applications. These methods include family-wise corrections (e.g., [5–9]), false discovery rates (e.g., [10–12]), and Bayesian approaches (e.g., [13–17]). Family-wise correction procedures are designed to test results from an entire study such that the probability of results from one or more test exceeding an experiment-wise significance threshold under the null hypothesis is not greater than a pre-specified level, α’. The use of false discovery rates restricts the expected proportion of false positive results across a study. Multiplicity problems in a Bayesian framework are usually handled by the use of an appropriately small prior probability density of alternative hypotheses. Storey showed that the posterior error probability resulting from Bayesian analyses is closely connected with the positive false discovery rate [17]. Other approaches have also been proposed including explicitly evaluating the tradeoff between type I and II error rates [18].

In this work, I present a decision theory based on an exchangeability principle to determine if adjusting for multiple tests is a necessary component of the statistical analysis or not. If the exchangeability principle is satisfied and an experiment-wise correction is employed, then, through relating properties of order statistics that reveal the null density of the top-ranked test, I develop a generalization of the Dunn-Šidàk correction which relaxes the assumption of uniformly-distributed nominal *p*-values under the null. Integration of this null generates the generalized Dunn-Šidàk experiment-wise *p*-value. As technological artifacts, cryptic relatedness, and population stratification produce departures from the uniform distribution of *p*-values under the null, this simple method may be particularly useful for genomic studies. Lastly, one can incorporate an effective number of tests procedure, such as the Cheverud-Nyholt method, into the generalized experiment-wise *p*-value calculation to treat the correlation between tests. Hence, the full approach enables the relaxation of both the uniform-distribution and independence assumptions in an experiment-wise correction.

## Methods

### Units of Analysis and Marker-Specific Hypotheses

Suppose that a case/control genetic association study is conducted to examine the correlation between genotypes and the presence of a disease. In such a study, genotypes are typically measured in cases and controls for a large number of single nucleotide polymorphisms (SNPs). Given data at a particular SNP, say ‘rs1’, there are many methods to quantify the evidence that rs1 is indeed associated with the disease studied, including the calculation of a *p*-value from the Armitage trend test or a Bayes factor. Now suppose that another SNP, rs2, is subsequently interrogated to again test for disease association with the same samples. Assume that the two SNPs are independent and independent conditioned on disease status. Should the evidence that rs1 is truly associated with the disease change after we have evaluated rs2? Stated this way, the common sense answer is no. By extension, the same reasoning applies to an arbitrary number of markers—the weight of evidence for rs1 being involved in the disease remains unaffected. Hence, if the hypothesis evaluated is specific to rs1, the probability of a significant disease association result for rs1 is independent of genetic information at other markers:

$$P(p_{rs1}<\alpha \text{|}rs2)=P(p_{rs1}<\alpha \text{|}rs2,\ldots ,rs(n))=P(p_{rs1}<\alpha )$$ (1)

where *p*_*rs*1_ is the *p*-value testing the null hypothesis of independence between genotypes at rs1 and disease status, α is a pre-specified significance level, and *n* indicates an arbitrary number of genetic markers. Therefore, when the hypothesis tested is framed with regard to rs1, the determination of disease association should be independent of the total number of markers evaluated—free from a standard family-wise type I adjustment procedure. Similarly, the statistical evaluation of rs1 should also not depend on the unit of how the result is reported: study-by-study. As biology cares not about how results are reported, much of human genetics work might fall under this statistical umbrella. Rather, for the unit of analysis when the hypotheses tested are specific to a marker, should be on a marker-by-marker basis. That said, on both theoretical (e.g., [19]) and empirical grounds (e.g., [20]), *α* = 0.05 appears to be wholly inadequate for striking a reasonable balance between type I and II error rates. Indeed, replication of findings with *p*>10^−6^ is quite infrequent [21]. This is particularly true if the proportion of markers across the genome having favorable disease mapping characteristics (i.e., genotype frequencies and disease effect sizes in which an average-sized study has at least moderate power to detect a significant result) is small. Under such a scenario—which is the prevailing view—an accounting for a very low prior probability that the individual marker evaluated is indeed disease-associated is necessary for proper statistical evaluation. It follows that tests of marker-specific hypotheses utilizing such a low prior probability regardless of the number of markers evaluated should be employed. The significance level for marker-specific hypotheses therefore becomes an objective metric that is not dependent on the parameters of a specific study and reporting of all markers evaluated is consistent with this framework. In a Fisherian/frequentist analysis, an appropriately reduced α could be employed based on the small prior, or in a Bayesian analysis a small prior could be explicitly used [22].

A natural extension of the marker-specific analysis paradigm would be the creation of informatics repositories of data at every marker evaluated in each study. Then (noting all of the caveats regarding meta-analyses across studies) evidence for or against each marker being involved in a particular phenotype can be amassed and compared against a stationary, objective significance level.

### An Exchangeability Principle for Multiplicity Testing

Now consider the situation where a large-scale genetic study, testing hypotheses that are not specific to particular markers—that is, *a priori*, the result from any marker may be reported as being significant. Under this scenario, the hypotheses tested need to be *a priori* exchangeable. Adjustment for multiplicity is therefore appropriate to account for the selection of top results from a larger set of tests. Under this framework, the top ranked test result is often of considerable interest. And since (i) the top-ranking could, *a priori*, be occupied by any marker and, (ii) if thought of successive tests within a study, there is clearly a non-zero probability that successive test results could occupy the top-ranked position, adjusting for the number of tests performed is appropriate.

### Use of Order Statistics in Experiment-wise Statistics

Assuming the exchangeability principle between markers as described above holds, warranting an accounting for multiplicity, the following uses order statistics to develop an experiment-wise statistical procedure which generalizes the Dunn-Šidàk method. This generalized method enables treatment of a broader set of empirically-defined null models using the distribution of p-values across a large-scale genetic study.

Suppose one has collected data from a large disease genetics study of *n* markers. For the development of the methods here, I will assume that all markers are in linkage equilibrium and independent conditioned on disease status. Let the null model (*H*_0_) consist of independence between disease status and genetics. Further assume that the proportion of tests that truly depart from the null is small relative to the proportion consistent with the null. For each marker, a nominal *p*-value is calculated testing the null hypothesis of independence with disease status, denoted by *p*. Provided large sample sizes, the absence of confounding, unrelated individuals, the condition that the number of null markers dramatically exceeds the number of true disease markers, and the appropriate nominal statistical methods, the density of the *p*-values is uniform on the unit interval. That is, *P*(*p* ≤ *α*|*H*_0_) = *α*. Let *U* = −*log*_*e*_(*p*). Under the null model described above, the density of *U* is simply the exponential: *f*(*u*) = *exp*(−*u*). Applying subscripts to indicate ranking with regard to calculated *p*-values yielding the ordering set:{*u*_1_, *u*_2,…,_ *u*_*n*_}, such that *u*_*n*_ is the largest value in the set of transformed *p*-values in the study, corresponding to the most significant marker. Hence, *u*_*i*_ indicates the *i*^*th*^ –ranked result from the set of *n* results. The general form for the density of the ranked *u*_*i*_ is

$$P\left(u_{i}\right)=i\left(\begin{array}{c} n\\ i \end{array}\right){\left[F\left(u\right)\right]}^{i-1}{\left[1-F\left(u\right)\right]}^{n-i}f\left(u\right);$$ (2)

where *F*(*u*) is the distribution function of *U*. This is a well-studied result from order statistics [23]. Hence, for the exponential density expected under the null,

$$\text{ence},\;P(u_{i})=i{(}_{i}^{n}){\left[1-exp(-u)\right]}^{\left(i-1\right)}{\left[exp(-u)\right]}^{\left(n-i+1\right)}$$ (3)

Under these assumptions, the probability density function (pdf) of the least significant test is the exponential

$$f(u_{1})=n{\left[exp(-u)\right]}^{n}$$ (4)

And the pdf of the most significant test is

$$f(u_{n})=n[exp(-u)]{\left[1-exp(-u)\right]}^{n-1}$$ (5)

Taking the result from Eq 5 as the null density, one can calculate an experiment-wise *p*-value, *p*_*E*,*n*_, for the nominal result for the most-significant −*log*_*e*_*p*. Let *x* denote this most significant result, negative log-transformed *p*-value.

$$p_{E,n}=n\int_{x}^{\infty }\left[exp(-u)\right]{\left[1-exp(-u)\right]}^{n-1}du$$

$$=1-{\left[1-exp(-u_{n})\right]}^{n},$$

which is simply the Dunn-Šidàk corrected *p*-value:

$$=1-{\left(1-p_{n}\right)}^{n},$$ (6)

This makes sense as we have derived the density of the top-ranked result under the null and calculated the rank-adjusted *p*-value in a standard manner assuming a uniform density for the *p*-values. For an arbitrary ranked result, the *p*-value accounting for rank is

$$p_{E,i}=i\left(\begin{array}{c} n\\ i \end{array}\right)\int_{x}^{\infty }{\left[1-exp\left(-u\right)\right]}^{i-1}{\left[exp\left(-u\right)\right]}^{n-i+1}du$$ (7)

### Generalization

The above derivation provides a useful framework for relaxing the original assumption of a uniform density for the nominal *p*-values. This carries broad utility as many studies have systematic biases or employ overly conservative or anti-conservative statistical techniques. For example, genome-wide association studies in human genetics can suffer from both problems: population stratification can generate systematic shifts in the *p*-value distribution through confounding, uncorrected effects from the inclusion of related individuals also generates departures from uniformly-distributed *p*-values under the null, and relying on limiting distributions for the calculation of *p*-values often changes the *p*-value distribution under the null. These systematic departures in the null *p*-value distribution from a uniform are problematic in the appropriate application of experiment-wise corrections. As shown above, the Dunn-Šidàk correction relies on strictly uniformly-distributed *p*-values under the null for an appropriate calculation of the top-ranked *p*-value accounting for multiplicity. By using an empirical approach assuming that the vast majority of markers are indeed consistent with the null model of no disease association, we can model these types of systematic departures in the null *p*-value distribution and incorporate these effects into a more general and realistic null model that focuses on the disease association. A similar approach was used for the calculation of Bayes factors in genome-wide association studies [24]. A natural generalization for the exponential distribution of *U* is a gamma density, of which exponentials are a special case:

$$f(u)={\left(\frac{u}{b}\right)}^{c-1}exp(-\frac{u}{b}){\left[bГ(c)\right]}^{-1}$$ (8)

The parameters *b* and *c* are the scale and shape parameters, respectively. Setting *c* = 1 restores the exponential distribution and setting both *b* = 1 and *c* = 1 yields the unit exponential variate analyzed above (for uniformly-distributed *p*-values). Applying the gamma distribution to Eq 2, one can calculate a rank-adjusted *p*-value under a more general null model. Focusing on the top-ranked result, the general ordered statistics result (setting *i* = *n* in Eq 2) is

$$P(u_{n})=n{\left[F(u)\right]}^{n-1}f(u)$$ (9)

Substituting the gamma distribution,

$$P(u_{n})=n{\left(\frac{u}{b}\right)}^{c}{\left[u\;exp(\frac{u}{b})Г(c)\right]}^{-1}{\left[1-\frac{Г(c,\frac{u}{b})}{Г(c)}\right]}^{n-1};$$ (10)

Where Г(*y*,*z*) is the incomplete gamma function equal to $\int_{z}^{\infty }t^{y-1}exp(-t)dt$. Integrating results in a generalized experiment-wise *p*-value for the top-ranked test,

$$p_{E,n}=\frac{n}{Г\left(c\right)}\int_{x}^{\infty }{\left(\frac{u}{b}\right)}^{c}{\left[u\;exp\left(\frac{u}{b}\right)\right]}^{-1}{\left[1-\frac{Г\left(c,\frac{u}{b}\right)}{Г\left(c\right)}\right]}^{n-1}du;$$ (11)

The parameters *b* and *c* can be estimated using the entire set of individual test −*log*_*e*_*p* values from the experiment or the subset of those values that is estimated to be consistent with the null hypothesis. Concurrent estimation of the gamma parameters is the subject of much work and both frequentist (e.g., [25]) and Bayesian approaches (e.g., [26]) have been studied. The matching moments estimators are easily derived using the arithmetic mean (*μ*) and sample variance (*σ*^2^) of {*u*_1_, *u*_2_,…,*u*_*n*_}:

$$\hat{b}\;=\;\frac{\sigma^{2}}{\mu }\text{ and }\hat{c}\;=\;\frac{\mu^{2}}{\sigma^{2}}$$

And maximum likelihood estimators are available [27]. Further, a goodness-of-fit test could also be employed to verify the utility of the gamma model (e.g., [28,29]. As the gamma parameters will be used to populate the density of negative log-transformed *p*-values, modeling a more general null, excluding the most significant test results from a large-scale study is a reasonable idea. More sophisticated approaches may be to concurrently model null and alternative densities as is often done with FDR procedures (e.g., [30]).

The general procedure outlined of using order statistics results to derive a null density for a top-ranked finding as a method for calculating experiment-wise *p*-values can be used in a number of scenarios. For example, one may want to model the *p*-value distribution as a Beta variate in which case the most significant finding, *q*, from *n* independent tests would have the null density of

$$n{\left[1-\;\int_{0}^{q}\frac{x^{v-1}{\left(1-x\right)}^{w-1}}{\beta \left(v,w\right)}dx\right]}^{n-1}\;\left[\frac{q^{v-1}{\left(1-q\right)}^{w-1}}{\beta \left(v,w\right)}\right];$$ (12)

where the parameters of the Beta variate are estimated from the observed set of *p*-values from the experiment. Another example would be to use the commonly-generated Chi-Squared test statistics from a large-scale experiment, modeled as either a Chi-Squared distribution or a Rayleigh-Rice distribution, and calculate the top-ranked density (Eq 13 below gives the Chi-Squared example),

$$n[2^{-v/2}c^{\left(v-2\right)/2}exp(-\frac{c}{2})]{\left[1-\frac{\Gamma (\frac{v}{2}\;,\;\;\;\frac{c}{2})}{\Gamma (\frac{v}{2})}\right]}^{n}{\left[\Gamma (\frac{v}{2})-\Gamma (\frac{v}{2},\;\frac{c}{2})\right]}^{-1}$$ (13)

Importantly, depending on the specifics of the application, the distribution selected to model the statistical measure (*p*-value, test statistic, or other metric) may have error that stems from the level of fit to observed data. That error would then propagate to the calculation of the experiment-wise *p*-values. Intuitively, the fit of the model to the tail of the distribution is highly critical to obtain accurate experiment-wise *p*-values through the methods promoted here.

### Treatment of Correlation among Tests

Thus far, the procedures outlined have developed a generalization of the Dunn-Šidàk correction, relaxing the assumption that *p*-values are uniformly-distributed under the null hypothesis. Still, the method assumes that the set of *p*-values are i.i.d., while the large majority of applications likely violate the independence assumption. Again, using the example from human genetics: with high-density genome-wide SNP/SNV panels and available sequencing technologies, sets of markers assessed for large-scale genetic studies exhibit widespread linkage disequilibrium. A simple way of incorporating the correlational structure of tests into the multiple testing calculation is through determining the effective number of independent tests [31–39]. Such approaches use the pairwise matrix of r^2^ values across all SNP/SNVs to perform the calculation and have been extended to include imputed data [35].

## Results

### Effect of Non-Uniform *p-*value Distributions

To explore the effect of a variety of different *p*-value distributions on the generalized multiplicity approach as compared to the Dunn-Šidàk correction, null-model *p*-values were generated as a beta variate in a Monte Carlo simulation. Given both shape parameters equal to unity, we recapitulate the uniform distribution for the null hypothesis, which is implicitly assumed in the Dunn-Šidàk procedure. As the shape parameters depart from unity, we can assess how this impacts both the Dunn-Šidàk and generalized method (making the Gamma model assumption) procedure. The proportion of top-ranked (most significant) tests that exceed a predefined significance level (α) are reported using both procedures. The Dunn-Šidàk corrected α is calculated according to Eq 6, whereas the generalized method corrected α is calculated by numerically solving Eq 11 for the upper limit of the integration such that the integral is equal to α. For example, if we model the *p*-value distribution in the Monte Carlo with a Beta variate having shape parameters ν = 0.9 and ω = 1.1, and simulate an experiment with 10,000 independent tests, we would calculate a generalized, Gamma-model significance level of 1.36E-06, corresponding to an experiment-wise α_G_ = 0.05. Whereas, one would calculate a Dunn-Šidàk level to be at a less conservative value of α_DS_ = 5.13E-06. The result of this difference in multiple test-adjusted significance levels is that, over 5,000 replications of this experiment, 17% of the top-ranked tests under the null exceed the α_DS_, but the Gamma model retains an appropriate significance level to control false positives with 5.2% of the top-ranked tests exceeding α_G_. Table 1 displays the results across 13 null models.

**Table 1:** Proportion of Top-Ranked Tests Exceeding Dunn-Šidàk and Gamma Model Significance Thresholds. The Dunn-Šidàk significance level (D-S alpha) is calculated in the traditional manner attempting to provide an experiment-wise significance level of 0.05. In an analogous fashion, the Gamma Model significance level (G alpha) is calculated using the simulated data, also attempting to generate an experiment-wise significance level of 0.05. Table 1 shows the results of a Monte Carlo simulation evaluating 13 models. Using the respective significance levels, the proportions of top-ranked tests exceeding the respective significance levels are presented.

Num Tests	ν	ω	D-S alpha	G alpha	Prop top ranked exceeding D-S	Prop top ranked exceeding G
**10,000**	**1**	**1**	**5.13E-06**	**5.13E-06**	**0.048**	**0.048**
**10,000**	**1.1**	**1.1**	**5.13E-06**	**1.57E-05**	**0.018**	**0.057**
**10,000**	**0.9**	**0.9**	**5.13E-06**	**1.30E-06**	**0.139**	**0.043**
**10,000**	**0.8**	**0.8**	**5.13E-06**	**2.06E-07**	**0.378**	**0.035**
**10,000**	**0.7**	**0.7**	**5.13E-06**	**2.01E-08**	**0.772**	**0.030**
**10,000**	**1.2**	**1.2**	**5.13E-06**	**3.79E-05**	**0.0074**	**0.065**
**10,000**	**0.9**	**1.1**	**5.13E-06**	**1.36E-06**	**0.170**	**0.052**
**10,000**	**0.8**	**1.2**	**5.13E-06**	**2.72E-07**	**0.506**	**0.057**
**10,000**	**0.7**	**1.3**	**5.13E-06**	**3.36E-08**	**0.910**	**0.073**
**10,000**	**1.1**	**0.9**	**5.13E-06**	**1.57E-05**	**0.012**	**0.047**
**10,000**	**1.2**	**0.8**	**5.13E-06**	**4.08E-05**	**0.0024**	**0.043**
**10,000**	**0.95**	**1.05**	**5.13E-06**	**2.68E-06**	**0.0924**	**0.048**
**10,000**	**1.05**	**0.95**	**5.13E-06**	**9.46E-06**	**0.0252**	**0.045**

### Example

**Fig 1:**
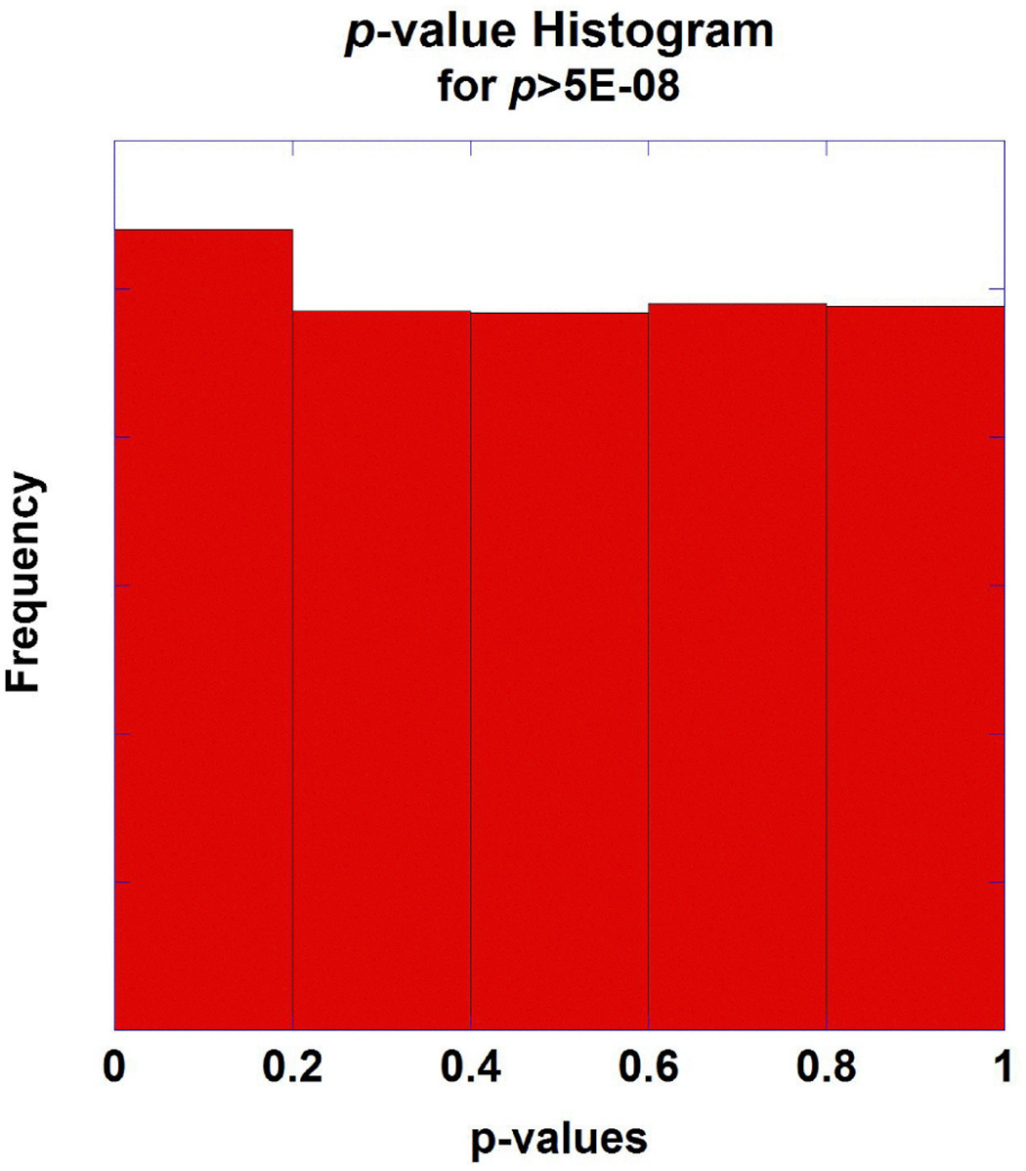
Histogram of Extreme BMI GWAS Association *p-*values. Fig 1 shows the histogram of the association *p*-values, adjusted for principal components, from the extreme BMI GWAS study.

**Fig 2:**
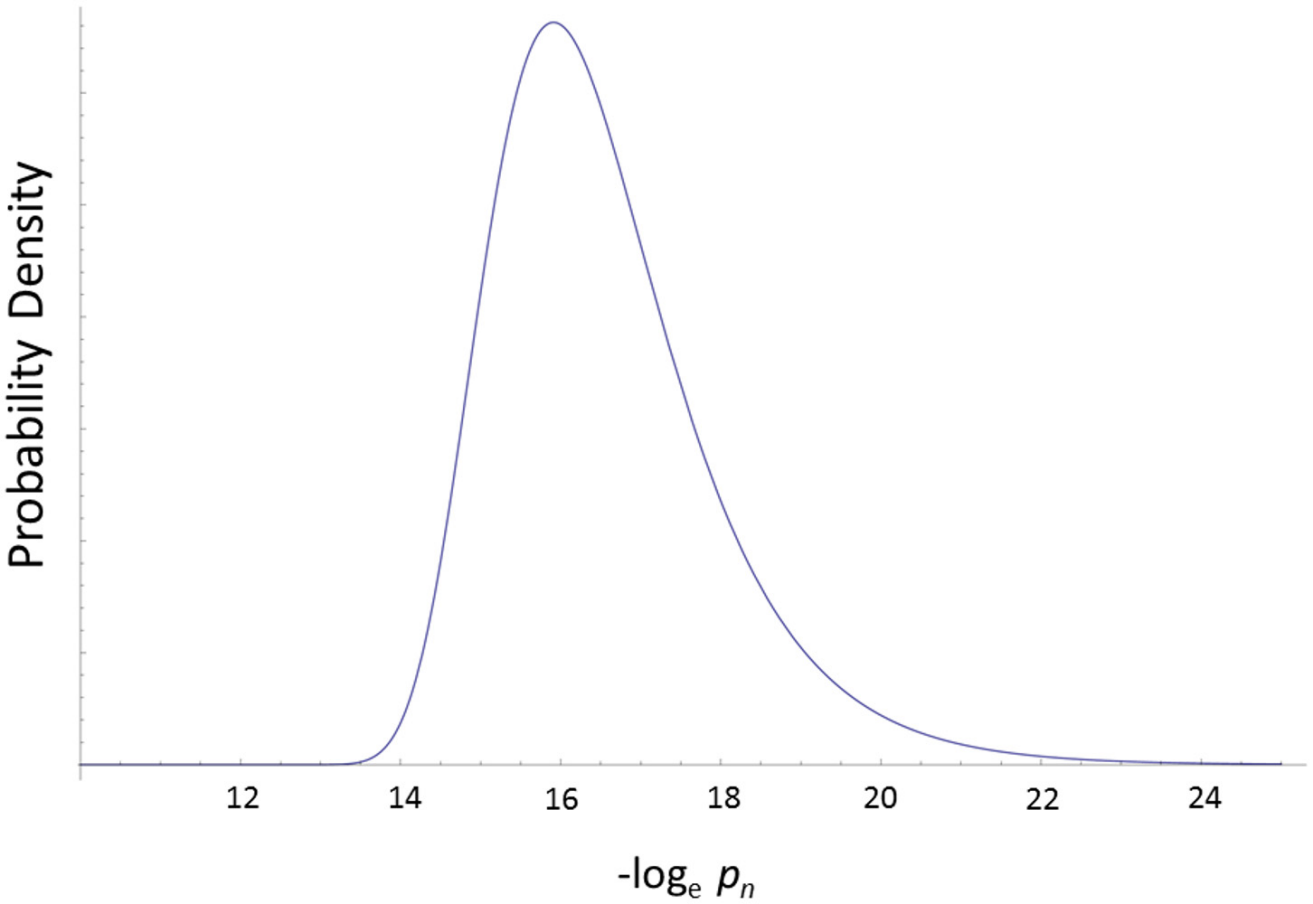
Probability Density for the Top-Ranked Marker under the Null Hypothesis. Fig 2 shows the null probability density function for the top-ranked marker from the extreme BMI GWAS using the gamma parameters.

To illustrate the use of this generalized family-wise approach to multiplicity, data from a genome-wide association study of extreme BMI was analyzed [40]. In this substantial study, 7962 cases were compared to 8106 controls and 1,984,813 SNPs were interrogated, generating nominal *p*-values from a model that was adjusted for principal components to correct for population stratification effects. The top-ranked SNP exhibited a *p* = 9.30E-33 (*x* = 73.7553) testing the association with extreme BMI. Not surprisingly, this SNP, rs11075990, is located within the well-established fat mass and obesity-associated gene, *FTO* on chr 16q12.2. To refine the list of *p*-values for the purpose of modeling a generalized null hypothesis, I removed 228 SNPs that exceeded the standard threshold for genome-wide significance (5.0E-08) and proceeded to estimate the parameters for the gamma distribution for the negative log-transformed *p*-values with the remaining 1,984,585 SNPs. Using matching moment estimators, $\hat{b}=1.1144$ and $\hat{c}=0.9334$. Recall, that under the uniform distribution for null *p*-values, both parameters should be unity. Fig 1 shows the histogram of *p*-values from this study following the removal of the 228 SNPs. Fig 2 shows the null density for the top-ranked marker in the extreme BMI study assuming independence between markers and utilizing the estimated, more general, gamma model. Integrating this null density of the top-ranked marker using Eq 11 to calculate an experiment-wise *p*-value, yields *p*_*E*,*n*_ = 2.601×10^−23^ for rs11075990 –highly significant by virtually any measure following adjustment for multiplicity. However, this is over a 1000-fold larger (less significant) than the Dunn-Šidàk corrected *p*-value of 1.85×10^−26^.

**Fig 3:**
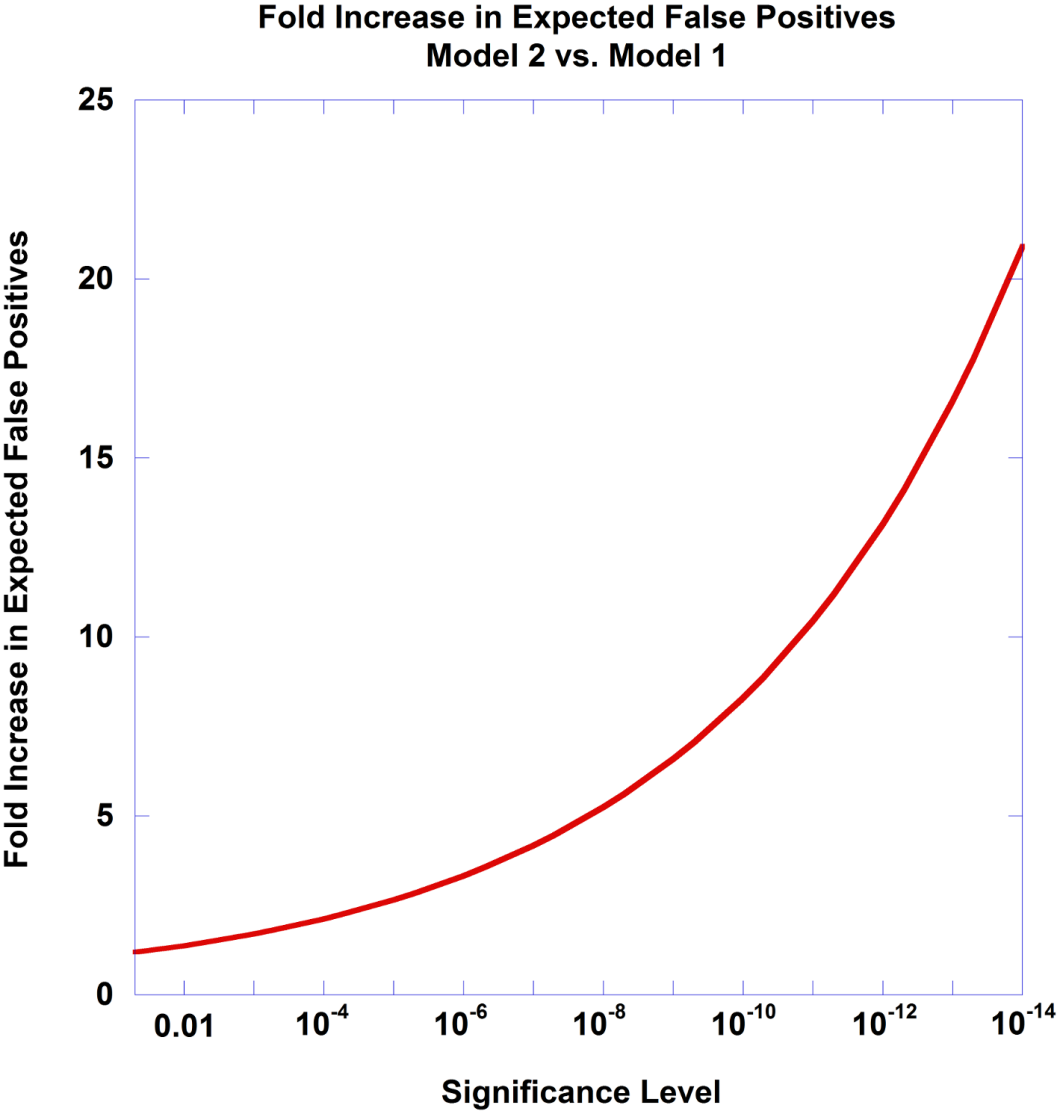
Fold Increase in Number of False Positive Tests for Model2 vs. Model1. Fig 3 displays the impact of mild departures in the null distribution of nominal *p*-values on the false positive rate. If one assumes the null *p*-value density is uniform (Model1; Gamma(1,1)) when in fact the null density is the given in Model2, Gamma(0.9334, 1.1144), using the significance levels as determined from Model1 will generate higher than expected false positive rates. The fold increase in this false positive inflation is plotted as a function of the significance level.

Comparing the fold increase in the rate of positive findings between the Gamma(1,1) model (Model1) and the Gamma(0.9334, 1.1144) model (Model2) as a function of α, we observe a substantial departure between the two models (Fig 3). For example, if we used a significance level of alpha = 1.0E-05 as determined from the null implicitly used in the Dunn-Šidàk correction (i.e., a uniform distribution of *p*-values), and we have measured 1,000,000 SNPs in a GWAS, then we would expect 10 positive findings under the null hypothesis. However, if the true null was defined by the Gamma model estimated above (Model2), then we would expect to see 26.6 false positive findings if we ignore this revised null in the determination of the significance level. Stated another way, if the real null distribution of *p*-values took on a Gamma model that was observed in the extreme BMI GWAS, then not adjusting for the effects of such a model (and implicitly using a uniform density for the Dunn-Šidàk correction) would produce an elevated number of false positives because the tail of the null distribution was inflated over that expected under a uniform distirbution. Such an increase in the number of false positive findings can greatly impact subsequent studies designed to replicate initial findings through genetic association or functional studies to investigate biological effects of variants in an associated region. Interestingly, analyzing a second GWAS, extreme height, from the same study [40], larger departures from a uniform distribution were found after filtering out all genome-wide significant tests ($\hat{b}=1.493$ and $\hat{c}=0.742$).

Treating the correlation among the tests will also change these experiment-wise *p*-values. Individual genotype data is not readily available from the Berndt et al study, so I will approximate the total number of independent tests using work from Gao X, et al. 2008 and Gao X, 2011 [34,35]. Gao [35], using the simpleM method, finds that using both measured and imputed SNPs (totaling 2.5M SNPs) from a set of Illumina beadchips applied to samples from the NHLBI Family Heart Study yields an effective number of independent tests of 540,818 markers (22.1% of the total number of tests evaluated). Similarly, Gao [34] shows that the estimated number of independent SNPs from the Illumina 1M Beadchip data was fairly similar: 413,360 markers. As a first-order approximation, we can take fit a line to these data and arrive at an estimated number of independent markers of 505,309 for the Berndt et al. dataset. Using this value, the Dunn-Šidàk corrected *p*-value is revised to 4.70×10^−27^. Similarly, revising the experiment-wise *p*-value gives *p*_*E*,*n*_ = 6.61×10^−24^, still with the same fold-increase (>1000-fold) in the *p*-value as with the previous calculation.

## Discussion

Several multiplicity ideas are presented here including (i) the importance of marker-specific hypotheses in large-scale genetic studies with the level of statistical significance sufficient for a positive result being free from the number of tests performed, (ii) the notion of exchangeability between tests as a criterion for use of multiple testing procedures, (iii) the connection between order statistics and the Dunn-Šidàk experiment-wise corrected *p*-value, and (iv) a more general framework for calculating an experiment-wise *p*-value when the assumptions of a strict null model may be mildly violated. The generalized experiment-wise multiplicity correction proposed uses the empirical data from large-scale experiments to account for departures from the uniform distribution of null *p*-values which are often generated through imperfect experimental designs, technological issues, sampling biases, and inaccurate statistical procedures.

## References

[1] Ioannidis JP, Ntzani EE, Trikalinos TA, Contopoulos-Ioannidis DG. Replication validity of genetic association studies. Nat Genet 2001; 29(3): 306–309. 11600885 10.1038/ng749

[2] Hirschhorn JN, Lohmueller K, Byrne E, Hirschhorn K. A comprehensive review of genetic association studies. Genet Med 2002; 4(2): 45–61. 11882781 10.1097/00125817-200203000-00002

[3] Gorlov IP, Moore JH, Peng B, Jin JL, Gorlova OY, Amos CI. SNP characteristics predict replication success in association studies. Hum Genet 2014; 133: 1477–1486. 10.1007/s00439-014-1493-6 25273843 PMC4384517

[4] Visscher PM, Brown MA, McCarthy MI, Yang J. Five years of GWAS discovery. Am J Hum Genet 2012; 90(1): 7–24. 10.1016/j.ajhg.2011.11.029 22243964 PMC3257326

[5] Bonferroni CE. Teoria statistica delle classi e calcolo delle probabilità. Pubblicazioni del R Istituto Superiore di Scienze Economiche e Commerciali di Firenze, 1936; 8: 1–62.

[6] Dunn OJ. Multiple comparisons among means. Journal of the American Statistical Association, 1961; 56(293): 52–64.

[7] Šidàk Z. Rectangular confidence regions for the means of multivariate normal distributions. Journal of the American Statistical Association, 1967; 62(318): 626–633.

[8] Dunn OJ. On multiple tests and confidence intervals. Communications in Statistics. 1974; 3(1): 101–103.

[9] Holm S. A simple sequentially rejective multiple test procedure. Scand J Statist 1979; 6: 65–70.

[10] Soric B. Statistical “discoveries” and effect-size estimation. J Am Stat Assoc 1989; 84: 608–610.

[11] Benjamini Y, Hochberg Y. Controlling the false discovery rate: a practical and powerful approach to multiple testing. *J R Statist Soc*. *B*. 1995; 57(1): 289–300.

[12] Storey JD, Tibshirani R. Statistical significance for genomewide studies. Proc Natl Acad Sci USA 2003; 100(16): 9440–9445. 12883005 10.1073/pnas.1530509100 PMC170937

[13] Scott JG, Berger JO. An exploration of aspects of Bayesian multiple testing. J Stat Plan Inference 2006; 136: 2144–2162.

[14] Wakefield J. Reporting and interpretation in genome-wide association studies. Int J Epidemiol. 2008; 37(3): 641–653. 10.1093/ije/dym257 18270206

[15] Efron B, Tibshirani R, Storey JD, Tusher V. Empirical Bayes analysis of a microarray experiment. J Am Stat Assoc 2001; 96: 1151–1160.

[16] Scott JG and Berger JO. An exploration of aspects of Bayesian multiple testing. J Stat Plan Inference 2006; 136: 2144–2162.

[17] Storey JD. The positive false discovery rate: a Bayesian interpretation and the q-value. Ann Stat 2003; 31(6): 2013–2035.

[18] Todorov AA, Rao DC. Trade-off between false positives and false negatives in the linkage analysis of complex traits. Genet Epidemiol 1997; 14: 453–464. 9358264 10.1002/(SICI)1098-2272(1997)14:5<453::AID-GEPI1>3.0.CO;2-2

[19] Sellke T, Bayarri MJ, Berger JO. Calibration of *p* values for testing precise null hypotheses. Am Stat 2001; 55: 62–71.

[20] Ioannidis JPA. Why most published research findings are false. PLoS Med 2005; 2: e124 16060722 10.1371/journal.pmed.0020124 PMC1182327

[21] Panagiotou OA, Ioannidis JPA. What should the genome-wide significance threshold be? Empirical replication of borderline genetic associations. Int J Epidemiol 2012; 41(1): 273–286. 10.1093/ije/dyr178 22253303

[22] Wakefield J. Commentary: Genome-wide significance thresholds via Bayes factors. Int J Epidemiol 2012; 41(1): 286–291. 10.1093/ije/dyr241 22345299 PMC3304534

[23] Feller W. An Introduction to Probability Theory and Its Applications. Vol. II, 2nd Ed John Wiley & Sons, New York; 1971.

[24] Schrodi SJ. A probabilistic approach to large-scale association scans: a semi-Bayesian method to detect disease-predisposing alleles. Stat Appl Genet Mol Biol 2005; 4: Article 31. 10.2202/1544-6115.1168 16646850

[25] Bowman KO and Shenton LR. Properties of estimators for the gamma distribution. Communications in Statistics B—Simulation and Computation. 1982; B11: 377–519.

[26] Son YS and Oh M. Bayes estimation of the two-parameter gamma distribution. Communications in Statistics—Simulation and Computation 2006; 35: 285–293.

[27] Evans M, Hastings N, Peacock B. Statistical distributions 2nd Ed John Wiley & Sons New York; 1993.

[28] D’Agostino RB and Stephens MA (Eds) Goodness-of-Fit Techniques. Marcel Dekker New York; 1986.

[29] Romantsova YV. On an asymptotic goodness-of-fit test for a two-parameter gamma-distribution. J of Mathematical Sci 1996; 81(4):2759.

[30] Storey JD. A direct approach to false discovery rates. J of Roy Stat Soc, Series B 2002; 64(3): 479–498.

[31] Cheverud JM. A simple correction for multiple comparisons in interval mapping genome scans. Heredity (Edinb) 2001; 87(Pt 1):52–58. 11678987 10.1046/j.1365-2540.2001.00901.x

[32] Nyholt DR. A simple correction for multiple testing for single-nucleotide polymorphisms in linkage disequilibrium with each other. Am J Hum Genet 2004; 74:765–769. 14997420 10.1086/383251 PMC1181954

[33] Li J, Ji L. Adjusting multiple testing in multilocus analyses using the eigenvalues of a correlation matrix. Heredity (Edinb) 2005; 95(3):221–227. 16077740 10.1038/sj.hdy.6800717

[34] Gao X, Starmer J, Martin ER. A multiple testing correction for genetic association studies using correlated single nucleotide polymorphisms. Genet Epidemol 2008; 32(4):361–369. 10.1002/gepi.20310 18271029

[35] Gao X. Multiple testing corrections for imputed SNPs. Genet Epidemol 2011; 35(3):154–158. 10.1002/gepi.20563 PMC3055936 21254223

[36] Moskvina V, Schmidt KM. On multiple-testing correction in genome-wide association studies. Genet Epidemiol 2008; 32:567–573. 10.1002/gepi.20331 18425821

[37] Dudbridge F and Gusnanto A. Estimation of significance thresholds for genomewide association scans. Genet Epidemiol 2008; 32:227–234. 10.1002/gepi.20297 18300295 PMC2573032

[38] Han B, Kang HM, Eskin E. Rapid and accurate multiple testing correction and power estimation for millions of correlated markers. PLoS Genet 2009; 5(4):e1000456 10.1371/journal.pgen.1000456 19381255 PMC2663787

[39] Chen Z and Liu Q. A new approach to account for the correlations among single nucleotide polymorphisms in genome-wide association studies. Hum Hered 2011; 72:1–9. 10.1159/000330135 21849789 PMC3171280

[40] Berndt SI, Gustafsson S, Mägi R, Ganna A, Wheeler E, Feitosa MF, et al Genome-wide meta-analysis identifies 11 new loci for anthropometric traits and provides insights into genetic architecture. Nat Genet 2013; 45: 501–512. 10.1038/ng.2606 23563607 PMC3973018

